# Relative Neurotoxicity of Ivermectin and Moxidectin in Mdr1ab (−/−) Mice and Effects on Mammalian GABA(A) Channel Activity

**DOI:** 10.1371/journal.pntd.0001883

**Published:** 2012-11-01

**Authors:** Cécile Ménez, Jean-François Sutra, Roger Prichard, Anne Lespine

**Affiliations:** 1 INRA, UMR1331, Toxalim, Research Centre in Food Toxicology, Toulouse, France; 2 Université de Toulouse, INP, UMR1331, Toxalim, Toulouse, France; 3 Institute of Parasitology, McGill University, Montreal, Canada; Swiss Tropical and Public Health Institute, Switzerland

## Abstract

The anthelmintics ivermectin (IVM) and moxidectin (MOX) display differences in toxicity in several host species. Entrance into the brain is restricted by the P-glycoprotein (P-gp) efflux transporter, while toxicity is mediated through the brain GABA(A) receptors. This study compared the toxicity of IVM and MOX *in vivo* and their interaction with GABA(A) receptors *in vitro*. Drug toxicity was assessed in Mdr1ab(−/−) mice P-gp-deficient after subcutaneous administration of increasing doses (0.11–2.0 and 0.23–12.9 µmol/kg for IVM and MOX in P-gp-deficient mice and half lethal doses (LD_50_) in wild-type mice). Survival was evaluated over 14-days. In Mdr1ab(−/−) mice, LD_50_ was 0.46 and 2.3 µmol/kg for IVM and MOX, respectively, demonstrating that MOX was less toxic than IVM. In P-gp-deficient mice, MOX had a lower brain-to-plasma concentration ratio and entered into the brain more slowly than IVM. The brain sublethal drug concentrations determined after administration of doses close to LD_50_ were, in Mdr1ab(−/−) and wild-type mice, respectively, 270 and 210 pmol/g for IVM and 830 and 740–1380 pmol/g for MOX, indicating that higher brain concentrations are required for MOX toxicity than IVM. In rat α1β2γ2 GABA channels expressed in *Xenopus* oocytes, IVM and MOX were both allosteric activators of the GABA-induced response. The Hill coefficient was 1.52±0.45 for IVM and 0.34±0.56 for MOX (p<0.001), while the maximum potentiation caused by IVM and MOX relative to GABA alone was 413.7±66.1 and 257.4±40.6%, respectively (p<0.05), showing that IVM causes a greater potentiation of GABA action on this receptor. Differences in the accumulation of IVM and MOX in the brain and in the interaction of IVM and MOX with GABA(A) receptors account for differences in neurotoxicity seen in intact and Mdr1-deficient animals. These differences in neurotoxicity of IVM and MOX are important in considering their use in humans.

## Author Summary

Ivermectin (IVM) is used for onchocerciasis mass drug administration and is important for control of lymphatic filariasis, strongyloidiases and Scarcoptes mange in humans. It is widely used for parasite control in livestock. Moxidectin (MOX) is being evaluated against *Onchocerca volvulus* in humans and is also widely used in veterinary medicine. Both anthelmintics are macrocyclic lactones (MLs) that act on ligand-gated chloride channels and share similar spectra of activity. Nevertheless, there are marked differences in their pharmacokinetics, pharmacodynamics and toxicity. Usually, both MLs are remarkably safe drugs. However, there are reports of severe adverse events to IVM, in some humans with high *Loa loa* burdens, and IVM can be neurotoxic in animals with defects in P-glycoproteins (P-gp) in the blood-brain barrier. We have compared the *in vivo* neurotoxicity of IVM and MOX in P-gp-deficient mice and their accumulation in brain. We also investigated their effects on mammalian GABA receptors. We show that MOX has a wider margin of safety than IVM, even when the blood-brain barrier function is impaired, and that the neurotoxicity *in vivo* is related to different effects of the drugs on GABA-gated channels. These observations contribute to understanding ML toxicity and open new perspectives for possible MOX use in humans.

## Introduction

Macrocyclic lactones (MLs) are a large family of broad spectrum antiparasitic drugs. Ivermectin (IVM), an avermectin macrocyclic lactone, is used in humans through mass drug administration programs for the control of onchocerciasis, a tropical parasitic disease caused by the filarial nematode *Onchocerca volvulus*. Moxidectin (MOX), a milbemycin (non-avermectin) macrocyclic lactone is currently being evaluated for possible use against *O. volvulus* in humans [1], [2]. Besides this, both drugs are commonly used in veterinary medicine in livestock to treat diseases caused by gastrointestinal nematodes and external parasites and for the prevention of *Dirofilaria immitis* infection in dogs.

In general, MLs have a high margin of safety in mammals (Pulliam & Preston, 1989). Indeed, P-glycoprotein (P-gp, *MDR1/ABCB1*), a plasma membrane efflux pump belonging to the ATP-binding cassette (ABC) transporters family, efficiently restricts their penetration in the brain at the blood–brain barrier [3], thus preventing their binding to the γ-aminobutyric acid type A (GABA(A)) receptor [4], [5]. However, neurotoxicity of IVM has been reported in mammals in cases of P-gp deficiency or overdose. In humans, IVM has been administered to tens of millions of individuals and is usually exceptionally safe when given at therapeutic doses [6]. Accumulation of the drug in the brain as a consequence of massive overdoses (more than 100 times the normal doses) is associated with prolonged coma and death [7], [8]. In addition, severe adverse events (SAEs) have been described after IVM treatment (0.15 mg/kg) in some individual humans carrying high burdens of the filarial nematode *Loa loa* [9], [10], [11], [12], [13] and IVM SAEs were recently associated with functionally relevant polymorphisms in human *MDR1* gene [14]. In 2005 and 2008, prior to initiation of MOX Phase II studies in humans infected with *O. volvulus* its safety was reviewed by WHO committees [http://whqlibdoc.who.int/publications/2008/9789241597333_eng.pdf, http://www.who.int/tdr/publications/tdr-research-publications/moxidectin/en/index.html] with conclusions that Phase II studies should proceed. Nevertheless, in contrast to the situation with IVM, MOX has, so far, only been administered to approximately 1,700 people in Phase I, II and III supervised clinical studies, without evidence of any serious adverse events [http://clinicaltrials.gov/ct2/show/NCT00856362, http://www.clinicaltrials.gov/ct2/show/NCT00300768, http://clinicaltrials.gov/ct2/show/NCT00790998].

IVM administrated at the therapeutic dose of 0.2 mg/kg to MDR1-deficient dogs provokes severe signs of neurotoxicosis including apparent depression, ataxia, somnolence and tremor [15], [16]. However, in dogs sensitive to 120 µg/kg IVM administered orally, a similar molar dose rate of MOX given by the same route did not produce any toxicological signs [17]. In another study, P-gp-deficient dogs that were sensitive to 120 µg/ml of IVM (20× the therapeutic dose rate (6 µg/kg) for IVM as a monthly heartworm preventative) did not produce signs of toxicosis following exposure to MOX at 100 µg/kg (more than the molar equivalent to 120 µg/ml IVM, and 33-fold the therapeutic dose rate (3 µg/kg) for MOX as a monthly heartworm preventative) given daily for 7 days [18]. In fact, MOX has been safely used on P-gp-deficient Collie dogs up to 32.5 mg/kg by spot-on application [19]. However, in addition to dose rate, route of administration can markedly affect toxicity and topical application is known to produce low bioavailability. In wild-type mice, the LD_50_ for oral administration of IVM is around 30 mg/kg [20] while the LD_50_ for MOX also given orally is 86 mg/kg [21].

**Figure 1:**
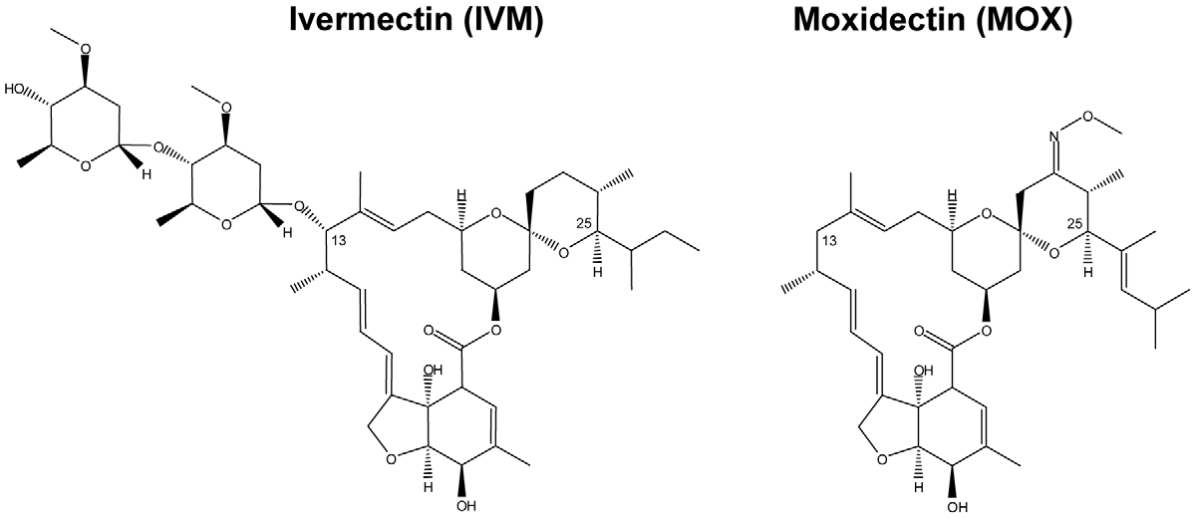
Comparison of chemical structures of ivermectin and moxidectin. Ivermectin is a mixture of B1a (substituent butyl on C25) and B1b (substituent isopropyl on C25) forms. The majority (more than 90%) of the drug is present as the B1a form.

Structural differences between MOX and IVM exist and include the absence of a disaccharide at position 13 of the macrocyclic ring in MOX, MOX being protonated (−H) at position 13, and the presence of a 23-methoxyimino group and other substitutions which distinguish it from IVM (Figure 1). These molecular differences presumably account for differences in their interaction with various invertebrate ligand-gated ion channels. Indeed, in *Caenorhabditis elegans* marked differences have been observed in the effects of IVM and MOX on pharyngeal pumping and motility (manifestations of the actions of these different MLs on ligand-gated chloride channels) [22]. Furthermore, difference in their interaction with mammalian ABC transporters has been demonstrated, MOX being much less (10-fold) effective than IVM (and other avermectins) in inhibiting transport activity by P-gp [23]. It is therefore reasonable to think that differences in drug interaction with mammalian GABA receptors could account for the differential toxicity of IVM and MOX.

While the interaction of IVM with mammalian GABA receptors has been known for some time, little is known about the interaction of MOX with these receptors and its potential CNS toxicity. In this context, the objectives of this work were (i) to compare the *in vivo* toxicity of MOX and IVM, (ii) to evaluate their accumulation in brain, and (iii) to compare their activity on the mammalian GABA(A) receptor. Given the major role of P-gp in the prevention of the penetration of MLs into the brain, acute toxicity *in vivo* and accumulation in the brain of these two MLs was assessed with Mdr1ab(−/−) knockout mice, deficient for the two P-gp murine isoforms, *Mdr1a* and *Mdr1b*.

## Materials and Methods

### Ethics statement

*In vivo* studies were conducted in mice under European laws on the protection of animals (86/609/EEC). Protocols are performed under procedure and principal for good clinical practice (CVMP/VICH 59598). The protocols for experimentation on rodents used in this manuscript have been approved by the local institutional animal care and ethics committee which is the “Direction Départementale des Services Vétérinaires de Haute-Garonne”. The specific approval number for this study approval is B31555-25.

### Materials

Ivermectin, γ-aminobutyric acid (GABA), collagenase type I, dimethyl sulfoxide (DMSO) and kanamycin solution (50 mg kanamycin/ml in 0.9% NaCl) were purchased from Sigma-Aldrich Chimie (St Quentin Fallavier, France). Moxidectin was a gift from Fort Dodge Animal Health. Penicillin-streptomycin solution (10,000 units/ml penicillin and 10,000 µg/ml of streptomycin) was obtained from Invitrogen - Life Technologies (Cergy Pontoise, France). All other chemicals were obtained from Sigma-Aldrich, unless otherwise stated. Rat GABA(A) α_1_, β_2_ and γ_2_ subunit constructs were a kind gift from Dr Erwin Sigel.

### Animal model

FVB Mdr1ab(−/−) mice, deficient for the two murine P-gps encoded by *abcb1a* and *abcb1b* genes (GenBankTM Accession numbers NM011076 and NM011075, respectively), were obtained from Taconic (NY, USA). Animals were kept under controlled temperature with a 12/12 h light/dark cycle. They received *ad libitum* a standard diet (Harlan Teklad TRM Rat/Mouse Diet; Harlan Teklad, Gannat, France) and municipal water. Mice were randomly assigned to groups and weighed. Experiments were carried out on 10–14 week-old mice (25–30 g).

### Drug administration in P-gp-deficient and wild-type mice for drug plasma and brain concentration measurement and for neurologic symptoms assessment

Suitable dilutions of a stock solution of IVM or MOX in DMSO were made in propylene glycol/formaldehyde (60∶40 v/v) for subcutaneous administration or in commercial formulations for oral administration in order to administer to each mouse the designated dose (µmol/kg body weight (bw)) in 100 µl. Each formulation was checked for drug concentration prior to administration.

For drug plasma concentration and brain accumulation assessment, Mdr1ab(−/−) mice were injected subcutaneously with an equivalent molar dose rate for both MLs in Mdr1ab(−/−) mice (6 animals per group): 0.23 µmol/kg, corresponding to 0.20 mg/kg and 0.15 mg/kg for IVM and MOX, respectively. Mice were sacrificed at 2 or 24 h after treatment.

To evaluate the plasma and brain concentrations of MLs as a function of the administrated dose, Mdr1ab(−/−) mice (6 per dose rate) were injected subcutaneously at various doses of IVM or MOX that were not lethal in 24 h: 0.1–0.3 mg/kg (0.114–0.342 µmol/kg bw) and 0.46 to 1.3 mg/kg bw (0.23–2 µmol/kg bw) respectively. In parallel, wild-type mice (3 per dose rate) were orally administered with doses close to their respective LD_50_: 20 and 25 mg/kg bw (22.8 and 28.6 µmol/kg bw) for IVM [20] and 18.3 and 40 mg/kg bw (28.6 and 62.5 µmol/kg bw) for MOX [21]. Mice were anesthetized 2 h or 24 h after administration with isoflurane and heparinized blood samples were collected from the orbital sinus vein. Immediately thereafter, mice were sacrificed by cervical dislocation and brains were rapidly removed. Blood samples were centrifuged at 1500 g for 10 min at 4°C and the plasma fraction was collected and stored at −20°C until analysis. The brains were washed in saline solution and frozen at −20°C until analysis.

For acute toxicity experiments, Mdr1ab(−/−) mice (2–8 per dose rate) were injected subcutaneously with drug solutions at dose rates ranging from 0.1–1.75 mg/kg bw (0.11–2.0 µmol/kg bw) for IVM and 0.2–8.2 mg/kg bw (0.31–12.9 µmol/kg bw) for MOX. Mice were observed for a period of 2 weeks and any neurological signs were recorded every 60 min for the first 12 hours and thence minimally twice per day. Mice were euthanized when severe tremors or ataxia were noted. The effective LD_50_ values (dose rate that caused 50% lethality) were determined graphically in Mdr1ab(−/−).

### Drug extraction and analytical procedures

ML concentrations were determined in plasma and brain by high performance liquid chromatography (HPLC) with fluorescence detection according to previously described and validated methods [24], [25]. In brief, plasma and tissues were homogenised in acetonitrile (1∶1 v/v or 1∶2 v/w, respectively). Samples were centrifuged at 2000 g and the supernatant applied to a Supelco C18 cartridge (Supelco Inc., Bellefonte, PA, USA) by using automated solid phase extraction (SPE). The extraction recoveries for the two molecules were 0.95 for plasma and 0.65 for brain. The eluate was evaporated and the dry extract was processed to obtain a fluorophore derivative by dissolving it in 1*N*-methylimidazole and trifluoroacetic anhydride solutions. Samples were injected into the HPLC system (PU980 pump, Jasko, Tokyo, Japan; 360 automatic injector, Kontron, Paris, France; RF-551 fluorescence detector, Shimadzu, Kyoto, Japan). For IVM and MOX a Supelcosil LC18 column (250×4.6 mm, 5 µm, Supelco, Bellefonte, PA, USA) was used with acetic acid (0.2% in water)∶methanol∶acetonitrile (4∶40∶56, v/v/v) as mobile phase.

### Expression of functional rat GABA(A) receptor in *Xenopus laevis* oocytes

Oocytes from *X. laevis*, injected with foreign cDNA of the receptor of choice, are a commonly used tool for studying the activity of plasma membrane receptors [26]. The cDNAs coding for the α_1_, β_2_ and γ_2_ subunits of the rat GABA(A) receptor channel have been described previously [27], [28]. cDNAs were dissolved in water and stored at −80°C. Isolation of oocytes from the frogs, defolliculation, culturing of the oocytes and injection of cRNA were performed as described previously [29]. Oocytes were injected with 46 nl of RNA solution, with RNA coding for α_1_, β_2_ and γ_2_ subunits at a ratio of 10∶10∶50 nM [30]. The injected oocytes were incubated in modified Barth's solution [90 mM NaCl, 3 mM KCl, 0.82 mM MgSO_4_, 0.41 mM CaCl_2_, 0.34 mM Ca(NO_3_)_2_, 100 U/ml penicillin, 100 µg/ml streptomycin and 100 µg/ml kanamycin, 5 mM HEPES pH 7.6] at 18°C for approximately 36 h before the measurements to ensure the expression of a functional receptor.

### Two-electrode voltage-clamp measurements

Electrophysiological experiments were performed by the two-electrode voltage-clamp method. Measurements were done in ND96 medium containing 96 mM NaCl, 2 mM KCl, 1 mM MgCl_2_, 1.8 mM CaCl_2_ and 5 mM HEPES, pH 7.5, at a holding potential of −80 mV. Currents were measured using a custom-made two-electrode voltage clamp amplifier in combination with an XY recorder (90% response time, 0.1 s). The intracellular electrodes were filled with 3 M KCl (resistance of 0.5–1.5 MΩ). Oocytes were exposed to ND96 or ND96 containing GABA with or without drugs by switching the perfusate using a ValveLink 8.2 perfusion system (AutoMate Scientific, Berkeley, CA). The perfusion solution (6 ml/min) was applied through a glass capillary with an inner diameter of 1.35 mm. GABA was prepared as a 10 mM stock solution dissolved in ND96. The modulatory compounds (IVM and MOX) were first dissolved in DMSO at 20 mM and then diluted in ND96 to the final concentration. The maximum concentration of DMSO used in perfusion was <0.01%, with application of DMSO alone at 0.1% not altering GABA responses.

Control concentration-response curves for GABA alone were obtained by perfusing the oocytes with a known GABA concentrations in ND96 saline until the maximal response (I_max_) was observed. To investigate the ability of MLs to potentiate the GABA-evoked current, oocytes were exposed to 2 µM GABA, which was responsible for approximately 10% of the maximal effect of the dose-response curve of GABA alone (EC_10_), followed by a 5 min recovery period. Subsequently, oocytes were exposed to a co-application of GABA (2 µM) with increasing ML concentrations. Concentration ranges used were: IVM: 0.5 nM–10 µM; MOX: 1 nM–5 µM. Relative current potentiation by MLs was determined as [(I _MLs+2 µM GABA_/I _2 µM GABA alone_)−1]×100 where I _2 µM GABA_ is the control current evoked by 2 µM GABA, I _MLs+2 µM GABA_ is the current evoked by each drug concentration in co-application with 2 µM GABA, and I_(MLs+2 µM GABA)Max_ is the maximal current evoked by co-application of drugs and 2 µM GABA.

A washout period of 5 min between each agonist application was introduced, allowing receptors to fully recover from desensitization. The perfusion system was cleaned, after application of MLs, between each experiment by washing with 10% DMSO in ND96 to avoid contamination. Three or four different batches of oocytes were used to collect data for each analysis.

### Data analysis and statistics

All experiments were conducted at least in triplicate. Differences in ML tissue concentrations were analyzed by one-way analysis of variance (ANOVA) with a Tukey post-test and are expressed as mean ± standard deviation (S.D.). GABA(A) receptor responses were plotted by least squares fit of log(agonist) versus response, with variable slope and are displayed as means ± standard errors (S.E.M.) (Prism 2.0, GraphPad Software Inc., San Diego, CA, USA). Half-maximal concentrations (EC_50_), slope factors (Hill coefficients, n_H_) and maximal potentiation values for ML-induced activation of current were obtained using the Hill equation. Statistical analysis was performed using unpaired t-tests with Welch's correction to allow for differences in variances (GraphPad Instat, San Diego, CA, USA). Differences were considered statistically significant when p<0.05.

## Results

### MLs-induced acute toxicity in Mdr1ab(−/−) mice

**Figure 2:**
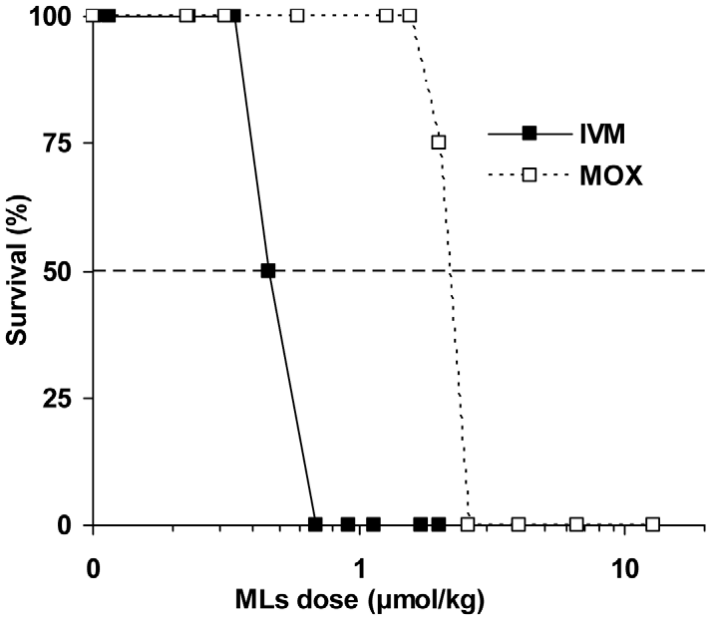
Acute toxicity of IVM and MOX in Mdr1ab(−/−) mice. Acute toxicity was determined by observing survival during a 14-day period after subcutaneous administration of IVM (black square) or MOX (open square) to small groups (2–8 animals) of Mdr1ab(−/−) mice. Extrapolation from the graph yields an estimated LD_50_ of 0.46 µmol/kg (0.40 mg/kg) and 2.3 µmol/kg (1.47 mg/kg) for IVM and MOX, respectively.

Acute toxicity of IVM and MOX was evaluated *in vivo* in P-gp-deficient mice. To determine the median lethal dose of these compounds, each drug was administered subcutaneously at increasing doses. The survival time of mice after the drug administration was recorded over two weeks, as well as the number of surviving mice in each group. Percent survival as a function of administered dose was plotted and the LD_50_ of each compound was determined graphically (Figure 2). Results show that the lethal dose for IVM was 0.46 µmol/kg (0.40 mg/kg), in good agreement with what was described previously [3]. The LD_50_ of MOX was 2.3 µmol/kg (1.47 mg/kg), a 5 times higher molar dose than that of IVM, demonstrating that MOX has much lower *in vivo* toxicity compared with IVM.

The behaviour of Mdr1ab(−/−) mice was observed following administration of both IVM and MOX and neurological signs are reported in Table 1. Upon administration of IVM at 0.11 µmol/kg no changes in the behaviour of mice were observed. At 0.46 µmol/kg of IVM, hyperactivity was observed just after administration and severe neurotoxic signs were observed 6 h post-administration. In contrast, MOX did not show any toxicity when administered at the same dose rate and rapid breathing was transiently observed 2 h post-administration of 2 µmol/kg of MOX. At dose rates close to the respective LD_50_ for IVM and MOX (0.46 and 2.3 µmol/kg), neurotoxic signs (lethargy, tremors and/or ataxia) of the drugs were observed starting at 6 h for IVM and 4 h for MOX (Table 1) and lethal toxicity occurred for these doses between 8 and 12 h.

**Table 1:** Neurological symptoms observed after IVM or MOX administration in Mdr1ab(−/−) mice.

		IVM			MOX	
Treatment (µmol/kg)	0.11	0.40^a^	0.69	2	2.6^b^	4
Time post- treatment						
1 h	Visually normal	Visually normal		Visually normal	Visually normal	Balance problems
2 h	″	″	Rapid breathing	Rapid breathing	″	Sleepiness
3 h	″	″		″	″	″
4 h	″	″		″	Sleepiness	″
5 h	″	″	Ataxia^c^	Visually normal	″	Ataxia^c^/Coma†
6 h	″	Lethargy	Lethargy†	″	″	
7 h	″	2/5 Ataxia^c^/tremor^d^ †		″	″	
8 h	″			″	″	
12 h	″	3/5 Sleepiness		″	Ataxia^c^/Tremor^d^ †	
1 d	″	Visually normal		″		
2 d	″	″		″		
14 d	″	″		″		

The Mdr1ab(−/−) mice (3 per dose rate) injected subcutaneously at dose rates of 0.11, 0.40 and 0.69 µmol/kg bw for IVM and 1.27, 1.64 and 2.56 µmol/kg bw for MOX were observed and the development of symptoms evoking neurologic signs (rapid breathing, balance problems, tremor, ataxia, sleepiness, lethargy) was recorded over a period of 2 weeks; every 60 min for the first 12 hours and thence minimally twice per day. Mice were euthanized when severe tremors or ataxia or profound lethargy was noted.

† Mice were euthanized when severe tremor or ataxia was noted.

a Dose rate averaging the LD_50_ for IVM.

b Dose rate averaging the LD_50_ for MOX.

c Ataxia: lack of voluntary coordination of muscle movements, as in walking.

d Tremor: rhythmic, muscle contraction and relaxation involving oscillations or twitching.

### ML accumulation in the brain

Because *in vivo* toxicity of IVM is known to be related to its entry into the central nervous system (CNS), we investigated the accumulation of the two MLs in the brain in order to identify whether IVM and MOX have a different propensity to access the CNS.

Plasma and brain concentrations of IVM and MOX were initially evaluated 2 and 24 h after a subcutaneous administration of each drug at an equimolar dose rate of 0.23 µmol/kg bw in Mdr1ab(−/−) mice. To compare the tendency of IVM and MOX to accumulate in the brain, the brain-to-plasma concentration ratios were calculated. Results are presented in Table 2. As expected and previously described [31], there was considerable accumulation of the two drugs in the brain tissue when P-gp was deficient; the brain concentration being even higher at 24 h that at 2 h. The brain-to-plasma concentration ratio for IVM was significantly higher compared with that for MOX, whatever the time studied (4.8±1.6 versus 2.2±0.7 ml/g, at 24 h, p<0.01), demonstrating that IVM has a greater ability to accumulate in brain tissue than MOX. Table 2 also shows that the plasma concentration at 24 h was significantly lower for IVM (22.0±8.1 pmol/ml) than for MOX (42.8±9.3 pmol/ml, p<0.001).

**Table 2:** Drug concentration in plasma and brain 2 and 24 h after SC administration of an equivalent molar dose rate of MLs in Mdr1ab(−/−) mice.

	Drug concentration in plasma (pmol/ml)		Drug concentration in brain (pmol/g)		Brain-to-plasma concentration ratio (ml/g)	
	2 h	24 h	2 h	24 h	2 h	24 h
***Ivermectin***	57.9±22.9	22.0±8.1	43.9±15.7	100.2±29.7	0.8±0.4	4.8±1.6
***Moxidectin***	60.0±14.5	42.8±9.3***	18.8±2.8*	93.0±28.9	0.3±0.1*	2.2±0.7**

Ivermectin (IVM) or moxidectin (MOX) was administered subcutaneously in Mdr1ab(−/−) mice (6 per drug) at similar molar dose rate (0.23 µmol/kg, corresponding to 0.20 mg/kg and 0.15 mg/kg for IVM and MOX, respectively). Mice were sacrificed at 2 or 24 h after treatment. Drug concentrations were determined in plasma and brain, and the brain/plasma concentration ratios were calculated.

* p<0.05,

** p<0.01,

*** p<0.001 *vs* IVM.

Interestingly, the IVM concentration in brain was more than 2-fold higher compared with MOX as early as 2 h after treatment (43.9±15.7 versus 18.8±2.8 pmol/g, p<0.05), demonstrating that IVM enters the brain more rapidly than MOX. When the drug concentration in brain was studied 24 h after administration, no significant difference was observed between the two compounds (100.2±29.7 and 93.0±28.9 pmol/g for IVM and MOX, respectively), showing that the overall brain exposure during a 24-h period did not significantly differ between IVM and MOX.

**Figure 3:**
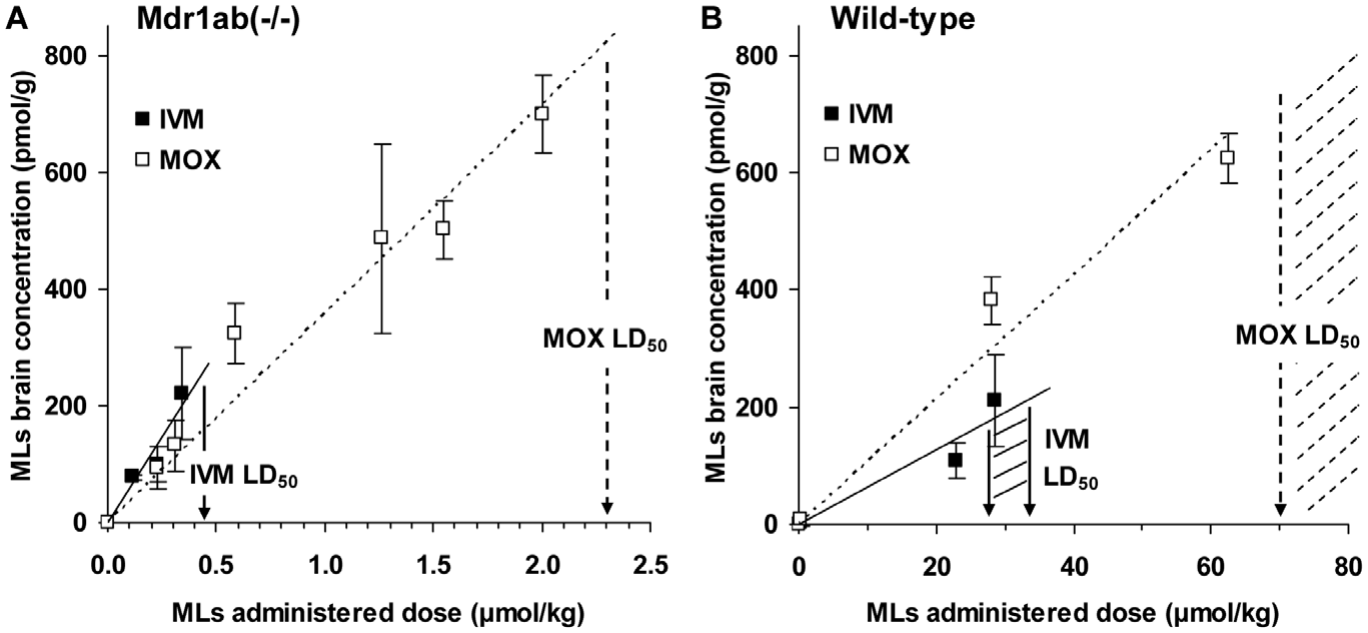
Absolute brain accumulation of MLs in Mdr1ab(−/−) and wild-type mice as a function of the administrated dose. IVM (filled squares) or MOX (empty squares) was administered to Mdr1ab(−/−) mice or to wild-type mice at increasing doses. Highest doses used for each ML were below the LD_50_ to ensure a non-lethal effect of the administration. Mice in each group were sacrificed 24 h after treatment and drug concentrations were determined in brain and plasma. Absolute brain accumulation was plotted against the administrated dose in (**A**) Mdr1ab(−/−) or (**B**) wild-type mice. A positive and significant linear correlation was observed between brain uptake and the administered dose rate (R^2^>0.94 in all cases). All measurements are expressed as mean ± S.D. of six animals.

Brain uptake of IVM and MOX was then evaluated in Mdr1ab(−/−) and in wild-type mice, 24 h after subcutaneous administration of increasing IVM and MOX dose rates. The highest dose rate used for this purpose was sublethal so that the relationship between drug concentration in brain and the *in vivo* neurotoxicity could be determined. The absolute brain level was plotted against the administered doses and the positive linear correlation between the brain uptake and the administered dose in Mdr1a(−/−) (Figure 3A) and in wild-type mice (Figure 3B) allowed us to calculate the absolute brain level that will be reached following administration of the drug at the LD_50_ value for each ML. These values were approximately 270 and 830 pmol/g in Mdr1ab(−/−) mice, and 210 and 740–1380 pmol/g in wild-type mice for IVM and MOX, respectively (Table 3). This demonstrated that MOX had to accumulate 3 times higher concentrations in the brain than IVM to provoke neurotoxicity in P-gp-deficient and in wild-type mice. Table 3 shows a positive and significant correlation between brain concentration and plasma concentration for both compounds in the two strains of mice. In Mdr1ab(−/−) mice, this linear relationship reveals that there was no drug brain saturation concentration within the doses studied, in accordance with the P-gp deficiency. The slope of the linear relationship, which reliably quantifies blood-brain transport, was 5.5 and 2.6 ml/g for IVM and MOX, respectively, demonstrating that 24 h after treatment, the brain-to-plasma concentration ratio for IVM was 2-fold higher than that for MOX, whatever the dose studied (Table 2). In wild-type mice, the brain-to-plasma ratio was considerably lower than in P-gp-deficient mice for both drugs (0.08 and 0.09 ml/g, respectively) in accordance with the presence of P-gp which limits the drug entrance into the brain.

**Table 3:** IVM and MOX concentrations in brain and brain-to-plasma ratio at increasing dose rates in Mdr1ab(−/−) and wild-type mice.

MLs	Mdr1ab(−/−)	Wild-type
***Ivermectin***		
LD_50_ (µmol/kg)	0.46^a^	27–34^b^
Brain concentration at LD_50_ (pmol/g)	270^c^	170–215^d^
Brain-to-plasma ratio (ml/g)	5.5 (R^2^ = 0.973)^e^	0.08 (R^2^ = 0.990)^e^
***Moxidectin***		
LD_50_ (µmol/kg)	2.3^a^	70–131^b^
Brain concentration at LD_50_ (pmol/g)	830^c^	740–1380^d^
Brain-to-plasma ratio (ml/g)	2.6 (R^2^ = 0.936)^e^	0.09 (R^2^ = 0.984)^e^

IVM or MOX was administered to Mdr1ab(−/−) mice (6 per dose rate) or to wild-type mice (3 per dose rate) at increasing doses below the LD_50_ to ensure a non-lethal effect of the administration. Drug concentrations were determined in brain and plasma after animals were sacrificed at 24 h post-treatment. Absolute brain accumulation was plotted against the plasma concentration to determine brain concentration at LD_50_, brain-to-plasma concentration ratio calculated and R^2^.

a LD_50_ determined graphically from Figure 1.

b LD_50_ for IVM and MOX determined from the literature [20], [21].

c Brain concentration reached at LD_50_, determined graphically from Figure 3A.

d Brain concentration reached at LD_50_, determined graphically from Figure 3B.

e Brain-to-plasma concentration ratio calculated and R^2^ obtained from the slope of the linear relationship between brain concentration and plasma concentration, which quantifies blood-brain transport.

### Measurement of GABA(A) channel activity by electrophysiological experiments

Differences between the toxicity of IVM and MOX could thus be related to a differential interaction of these two compounds with brain GABA receptors. We therefore compared the ability of IVM and MOX to activate GABA(A) receptors expressed in *Xenopus laevis* oocytes.

#### Effect of the reference agonist GABA alone

**Figure 4:**
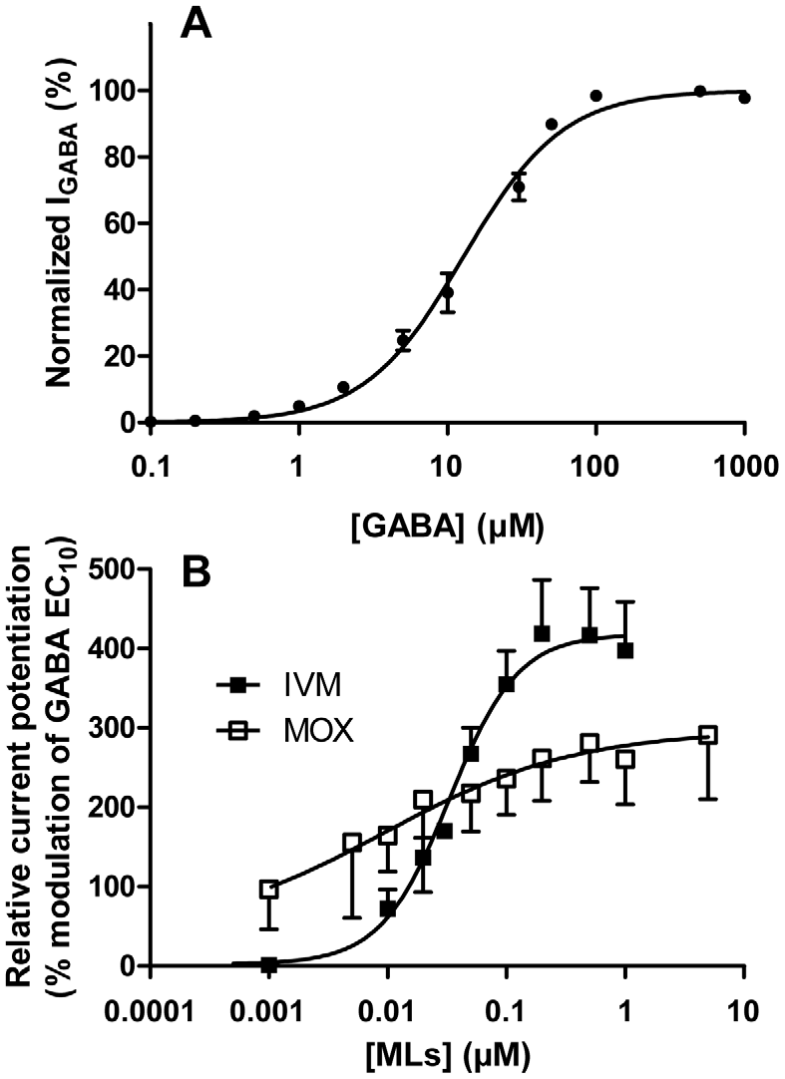
Concentration-response curves of rat GABA(A) receptor expressed in *Xenopus* oocytes. (**A**) Average concentration-response curve for the reference agonist GABA alone. Data were normalized to the maximum GABA-evoked response and fitted to the Hill equation (EC_50_ = 12.8±0.3 µM, Hill slope = 1.30±0.02. Data are given as mean ± S.E.M. from 3 independent oocytes batches (n = 4 oocytes for each batch). (**B**) Concentration-dependent potentiation of the GABA receptor, presented as the percentage of the GABA-evoked response at EC_10_ (2 µM). To analyse the potentiation of the GABA-evoked current induced by IVM or MOX, GABA-responsive oocytes were exposed to 2 µM GABA, followed by washing and then 2 µM GABA in association with increasing concentrations of IVM (n = 8) or MOX (n = 5). Data were fitted to the Hill equation and are given as mean ± S.E.M.

Oocytes expressing rat α_1_β_2_γ_2_ GABA_A_ receptors were exposed to saline containing 0.05 µM–1 mM GABA. GABA-induced inward Cl^−^ currents were normalized to the maximum GABA-induced current (100 µM) and plotted against the GABA concentration to obtain a concentration–response curve for GABA according to the Hill equation. The average curve for GABA is presented in Figure 4A. This curve was used to determine the effective concentration EC_10_ value, i.e., the concentration producing 10% of maximal response, which amounted to 2.08±1.18 µM. The EC_50_ and Hill slope were also determined using the Hill equation and amounted to respectively 12.84±1.06 µM GABA and 1.30±0.07 (n = 12). These values were comparable with previous reports [29], [30], [32], [33].

#### Effects of IVM and MOX on the GABA-induced response

The effects of IVM and MOX on GABA-induced membrane currents were then examined. The MLs-mediated potentiation of the current was measured at a concentration of 2 µM GABA, which elicits only a small fraction of the maximal current amplitude (∼EC_10_, Figure 4A). Increasing concentrations of MLs were co-applied with 2 µM GABA to determine their concentration-response curves relative to the GABA response. The currents elicited by MLs in association with GABA were expressed as a percentage of the maximal potentiated current elicited by co-application of each drug with 2 µM GABA.

At low receptor occupancy (EC_10_), IVM and MOX were both able to potentiate the GABA-induced response, demonstrating that IVM and MOX can act as allosteric modulators on the mammalian GABA(A) receptor when co-applied with GABA at EC_10_. The concentration-response relationship of the stimulation of GABA-evoked current of rat GABA(A) receptor expressed in *Xenopus* oocytes by IVM and MOX, normalized to the maximum GABA-evoked response and fitted to the Hill equation, is presented in Figure 4B. Clear differences are observed in the shape of the GABA-potentiation curve by MOX and IVM. The analysis of the curves revealed that at a concentration averaging 0.5 µM IVM caused almost twice the maximal potentiation of the GABA(A) receptor compared with MOX (413.7±66.1 versus 257.4±40.6%, respectively, p<0.05) (Table 4). The slope of the curve was clearly shallower for MOX than for IVM. This was confirmed by the Hill coefficient values which are significantly different (p<0.001, Table 4). These slope factors, which give information about the steepness of the dose-response plot, were 1.52±0.45 and 0.34±0.56 for IVM and MOX, respectively. This result shows that the potentiating response to increasing concentrations of IVM, on opening the GABA channel, is much greater that for MOX.

**Table 4:** ML modulation of the GABA-gated currents.

MLs	EC_50_ (nM)^a^	Hill slope	Concentration to reach E_max_ (µM)	E_max_ (%)^b^
***Ivermectin (n = 8)***	33.8±7.7	1.52±0.45	0.49	413.7±66.1
***Moxidectin (n = 5)***	11.8±9.2	0.34±0.56***	0.66^c^	257.4±40.6* c

a Concentration that evoked 50% of the maximal response for each ML.

b Maximum potentiation relative to GABA alone.

c n = 4 for calculation of concentration to reach E_max_ and E_max_ (%) for MOX, because for one egg, it was not possible to calculate the MOX E_max_ (%) from the fitted curve.

* p<0.05 vs IVM;

*** p<0.001 *vs* IVM.

## Discussion

It is now well recognised that MOX is less toxic than IVM in some invertebrate species, such as dung beetles [34] and the Anopheles mosquito [35], and in some mammals, such as wild-type mice [20] and in collie dogs sensitive to IVM. Although this information is important in the context of optimizing the use of MLs in humans and animal parasite control, the mechanisms for such differences remain unknown. The differential toxicity of the avermectins and MOX suggest several hypotheses: (i) ML compounds are transported differently across the blood–brain barrier (BBB), (ii) they accumulate to a different extent in the CNS tissue leading to a different drug concentration arriving at the target and/or (iii) they have different effects on vertebrate CNS receptors. We therefore compared the *in vivo* neurotoxicity of IVM and MOX in mice, their accumulation in the brain tissue and their ability to potentiate the mammalian GABA(A) receptor, expressed in *Xenopus* oocytes. Given that IVM and MOX are differentially transported by P-gp [31], this study was performed with P-gp-deficient Mdr1ab(−/−) mice, in order to remove any P-gp contribution to the entry of the drugs into the brain.

In this study, first neurotoxicity signs were observed at lower doses for IVM compared with MOX. Mdr1ab(−/−) mice were found to be approximately 5-fold more sensitive to subcutaneous administered IVM than to MOX with an LD_50_ of 0.46 µmol/kg bw for IVM and of 2.3 µmol/kg bw for MOX (p<0.01). This result is consistent with previous work reporting an IVM LD_50_ of 0.6–0.8 µmol/kg bw in Mdr1a-deficient mice which had been mutated on only the *abcb1a* gene and had a compensatory increase in expression of the *abcb1b* gene [3]. Further, our results in the Mdr1ab−/− mice are consistent with observations that MOX did not induce any signs of toxicosis in IVM-sensitive dogs, which are P-gp-deficient [17], [19].

The first hypothesis to explain this different drug tolerance is that IVM and MOX are differentially transported across the BBB *in vivo* and levels of accumulation of these MLs differ in the brain [36]. In the present study, the brain-to-plasma concentration ratio following subcutaneous administration of 0.23 µmol/kg MLs in P-gp-deficient mice was more than 2-fold higher for IVM than for MOX (4.8±1.6 ml/g and 2.2±0.7 ml/g at 24 h for IVM and MOX, respectively, p<0.01). Moreover, when increasing doses were administered, the slope of the brain tissue concentration versus plasma concentration curve was higher for IVM than for MOX (Table 3). These results are in accordance with previous data [31], demonstrating that in the absence of P-gp IVM has a higher penetration rate into the brain tissue than MOX.

MOX has a higher lipophilicity than IVM (logP _MOX_ = 6; logP _IVM_ = 4.8), and one would expect a higher affinity of MOX for this tissue. This led to the hypothesis that other drug efflux transporters at the BBB besides P-glycoprotein [37], could limit the entry of MOX but not that of IVM into brain. Knowing that the defective P-gp in the mutant Mdr1a(−/−) mice was associated with increased *Abcg2* mRNA, encoding the efflux transporter Bcrp, at the BBB [38], this hypothesis is in agreement with a recent study where MOX was identified as a BCRP substrate [39].

In most animals, MOX has a longer plasma half-life than IVM [39], and the pharmacokinetics of MOX have recently been studied in humans [40], [41], [42], [43]. The difference in half-life between IVM and MOX may alter the kinetics of accumulation of MOX relative to IVM in brain and the time of maximal concentration of MOX and IVM in this tissue. Interestingly, we showed in this study that the absolute level of drug accumulation in the brain in Mdr1ab(−/−) mice, at the LD_50_ dose rate was more than 3-fold higher for MOX than for IVM (830 versus 270 pmol/g, respectively). These concentrations were very similar to those measured in the brain of wild-type mice after administration of their corresponding LD_50_. These data demonstrate that neurotoxicity of a ML compound is not strictly related to its ability to enter and accumulate in the brain.

Another hypothesis to explain differences between the toxicity of IVM and MOX could thus be related to a differential interaction of these two compounds with GABA receptors in the brain or any tissues where GABA receptors are localized such as the enteric nervous system and sympathetic ganglia. We have shown here on the rat α_1_β_2_γ_2_ GABAergic Cl^−^ channel that both IVM and MOX were able to activate and potentiate the currents elicited by the reference agonist GABA, in a concentration-dependent manner. This potentiating effect was observed when receptor occupancy was low, i.e. when co-applied with GABA at its EC_10_ (allosteric effects are commonly assessed at agonist concentrations between the EC_10_ and EC_20_ for the agonist), demonstrating that IVM and MOX act as allosteric modulators of the mammalian GABA(A) receptor. This is supported by the observation that when GABA was co-applied with MLs in sub-saturating concentrations, the amplitude and time course of the elicited current were higher than when GABA was applied alone. However, the rate for dissociation of IVM and MOX from the receptor was much slower than that for GABA (data not shown), indicating allosteric binding site(s) for the MLs on the receptor could exist. Indeed, in accordance with our results, IVM was known to interact with GABAergic receptors and was previously shown to potentiate the GABA-elicited currents of chick neuronal GABA-gated chloride channels [29], and also of a nematode putative GABA(A) receptor [44], [45]. Previous binding studies have led to the conclusion that IVM binds in a two step process, on the GABAergic receptor resulting in activation of the receptor after binding to a high affinity site and blocking it on further binding to a low-affinity site [5].

As far as MOX was concerned, direct evidence for its interaction with mammalian GABA receptor channels has been missing so far. It has only been reported that MOX, like IVM, blocked binding of 4-n-[3H]propyl-4′-ethynylbicycloorthobenzoate to GABA receptors in *Drosophila melanogaster* [46]. Here, we clearly demonstrate for the first time that MOX was able to potentiate the GABA-activated currents mediated by rat α_1_β_2_γ_2_ GABA(A) receptors and the ability to potentiate GABA action is different between IVM and MOX. MOX had a non significant lower EC_50_ compared with IVM (p = 0.054). Interestingly, it has been demonstrated that both MOX and IVM bind the *Cooperia oncophora* glutamate-gated chloride channel GluClα3, expressed in *Xenopus* oocytes, and the EC_50_ of the MOX for opening the receptor in the presence of the natural ligand was lower compared with IVM [47].

Of considerable interest was our finding of differences in the shape of the GABA-potentiation curves, revealing differences in the actions of the two compounds. The Hill coefficient, which reflects the steepness of the dose-response plot, was 1.52 for IVM and only 0.34 for MOX. A Hill coefficient greater than one for IVM suggests positive cooperativity, i.e., once one molecule is bound to the receptor, the affinity of the receptor for the molecule increases [48].

A Hill coefficient lower than one, found for MOX, suggests a negative cooperativity. Furthermore, IVM caused an almost 2-fold maximum potentiation of the GABA(A) receptor compared with MOX.

These data clearly indicate that at a sublethal concentration of IVM in brain (270 pmol/g corresponding to 0.27 µM which is close to the concentration for maximal effect and is approximately 8× higher than the IVM EC_50_ for the potentiation of the GABA channel), IVM would have a greater potentiation of GABA action on this receptor than would MOX at asimilar brain concentration. Therefore, as IVM concentrations increase, it may potentiate the effects of GABA binding and opening the channel to a much greater extent than will MOX at similar elevated concentrations (Figure 4B), with consequences for depolarization of neurons expressing GABA(A) receptors and neurotoxicity. We propose that this is the cause of the higher toxicity of IVM when compared with MOX when ML concentrations increase in the brain.

The differences seen in receptor activation between IVM and MOX might be related to a difference in the structure of the MLs. Indeed, differences between IVM and MOX in the case of their interactions with mammalian ABC transporters, especially with P-gp, have already been demonstrated [23]. Moreover, a model for the IVM binding site and atomic interactions with amino acids in a *C. elegans* glutamate-gated chloride channel have recently been proposed [49]. Considering the structural differences between IVM and MOX, i.e., absence of the disaccharide moiety on the C-13 of the macrocycle, a methoxime moiety at C-23 and an olefinic side chain at C-25, it has also been postulated that the interaction of MOX with the glutamate-gated chloride channel will be different from that of IVM [50]. It is therefore reasonable to expect that MOX may also interact differently from IVM on GABA-gated chloride channels.

In addition to interaction with GABA(A) receptors in the brain, it has been shown that IVM potentiates purinergic (ATP) P3X (cationic) receptors [51] and acetylcholine receptors [52] in the brain. However, potentiating these receptors requires relatively high concentrations of IVM, 3 and 30 µM, respectively, which is considerably higher than the concentrations that markedly potentiated the GABA receptor. Nevertheless, IVM and MOX could exert some of their neurotoxicity via receptors in the brain other than GABA(A) receptors and this needs further investigation.

Differences in the accumulation of IVM and MOX in the brain, in the role of the P-gp transporter in the BBB and in the interaction of IVM and MOX with GABA(A) receptors in the brain may account for differences in neurotoxicity seen in intact and P-gp-deficient animals. These differences in neurotoxicity of IVM and MOX may be important in considering their use in humans.

In summary, we have demonstrated *in vivo* that in the case of P-gp deficiency (i) IVM has a higher penetration rate into the brain whatever the dose administered and enters the brain more quickly than MOX and (ii) the brain uptake threshold value leading to neurotoxicity is lower for IVM than for MOX. In addition, we have shown for the first time, that *in vitro*, MOX can interact with the mammalian GABA(A) receptor as an allosteric modulator by enhancing the actions of GABA. Our data indicate that MOX at high brain concentrations is less efficient in potentiating GABA-mediated opening of the GABA(A) receptor than is IVM. Altogether these data show that MOX has a wider margin of safety than IVM, even when the BBB function is impaired. These observations contribute to understanding ML-induced toxicity and open new perspectives for using MOX in humans.
